# Supplementary material for: V1-bypassing suppression leads to direction-specific microsaccade modulation in visual coding and perception
Source: Nat Commun. 2022 Oct 26;13:6366. doi: 10.1038/s41467-022-34057-3 (PMC9606005; doi:10.1038/s41467-022-34057-3)
Supplement: Supplementary file 1 — Supplementary Information [file 41467_2022_34057_MOESM1_ESM.pdf]

V1-bypassing Suppression Leads to Direction-Specific Microsaccade Modulation in Visual Coding and Perception

Supplemental Information

Supplementary Figures

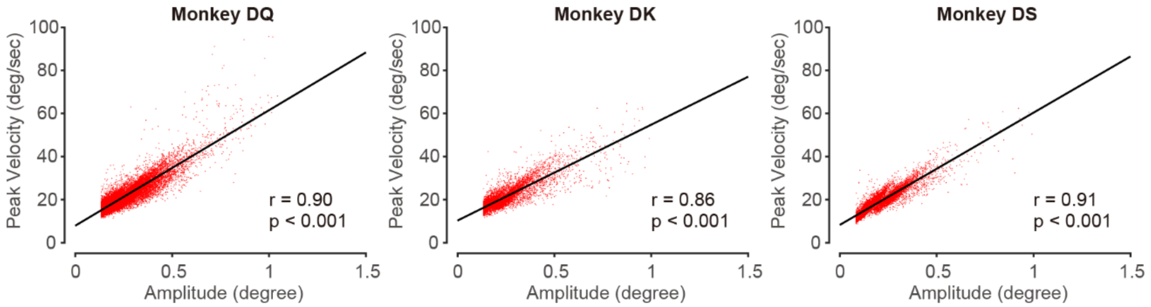

Supplementary Fig. 1 Correlation between peak velocity and amplitude of microsaccades in three monkeys

Peak velocity and amplitude for each microsaccade are highly correlated in all three monkeys (DQ: Pearson's correlation coefficient $r = 0.90$, $p < 10^{-40}$; DK: Pearson's correlation coefficient $r = 0.86$, $p < 10^{-40}$; DS: Pearson's correlation coefficient $r = 0.91$, $p < 10^{-40}$).

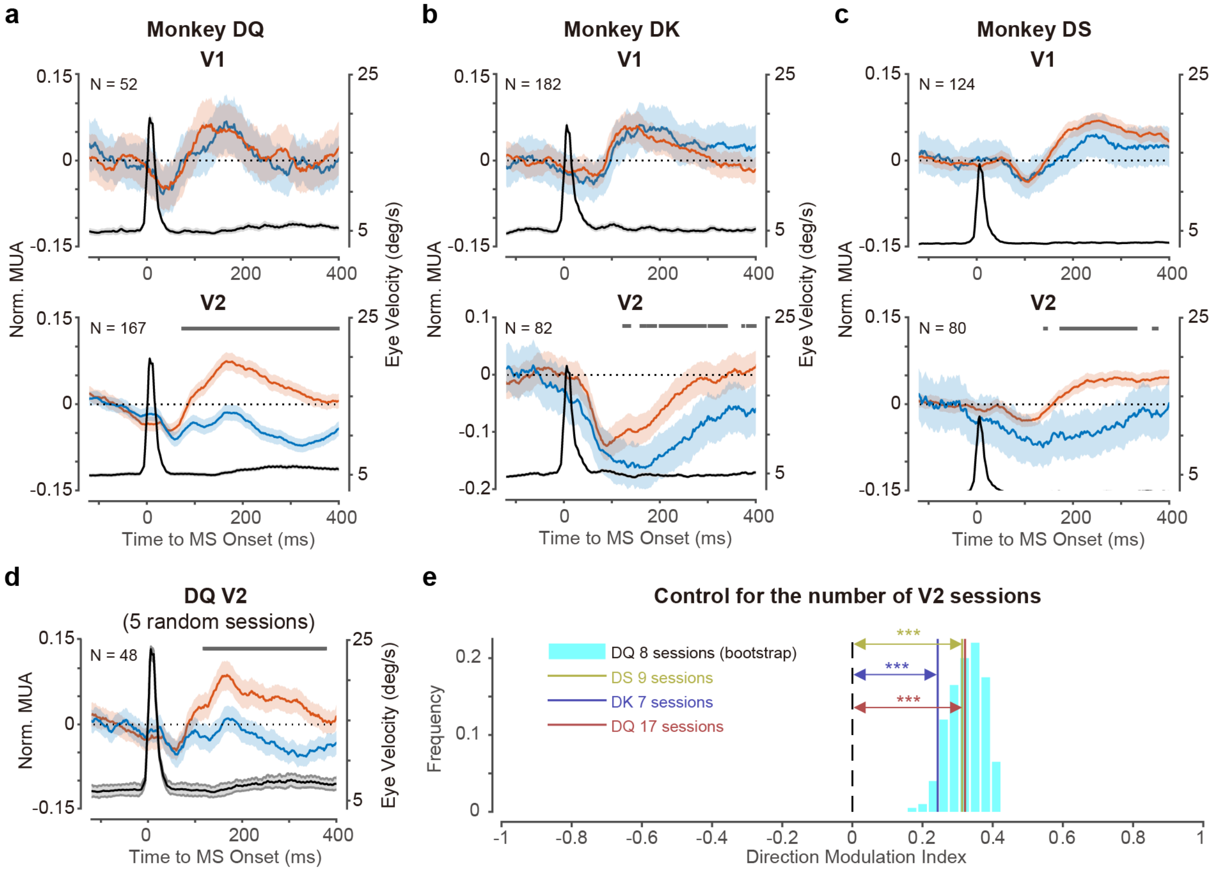

Supplementary Fig. 2 Microsaccade modulation in V1 and V2 in three monkeys

a-c Normalized MUA (mean ± s.e., orange and blue lines in the left vertical axis) in V1 (top) and V2 (down) and averaged velocity of eye movement (the black line in the right vertical axis) around microsaccade onset in monkey DQ (**a**), DK (**b**) or DS (**c**). Gray bars on the top indicate the significant time period (two-sided paired *t*-test with Bonferroni corrections). **d** Normalized MUA and velocity of eye movement (mean ± s.e.) from 5 randomly selected session of monkey V2 recordings. **e** Distribution of means of direction modulation index (DMI) for bootstrapped sessions (n = 200). All means of subsets from DQ V2 data were above zero, indicating a consistent and robust directional modulation in V2 of DQ. Vertical solid lines represent the means of DMI for all sessions from DS (yellow) DK (blue) and DQ (red). Asterisks donates statistical significance (two-sided t test, $^{***}p < 10^{-4}$, DQ: $p = 4.17\times10^{-37}$; DK: $p = 3.07\times10^{-5}$; DS: $p = 1.64\times10^{-7}$).

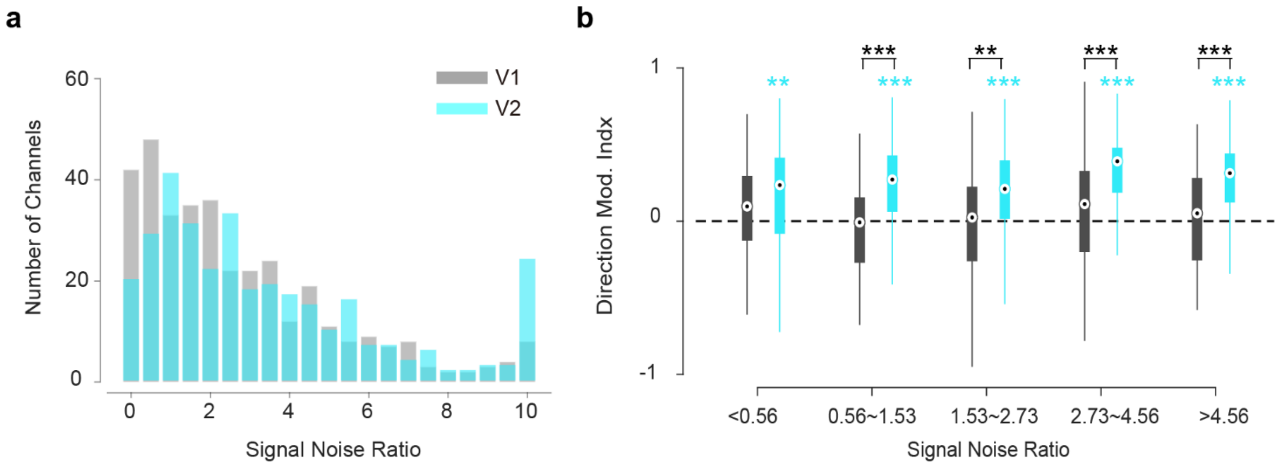

Supplementary Fig. 3 Direction modulation index of V1 and V2 was consistent in different levels of signal noise ratio

a Distribution of signal noise ratio for all units in V1 and V2. **b** Box plots show the median (middle points), 25th, 75th percentile (box) and 1.5 interquartile range (whiskers) of direction modulation index of V1 (n = 358 sites) and V2 populations (n = 329 sites) in SNR groups defined by quintiles. '**' indicates $p < 0.001$ and '***' indicates $p < 10^{-5}$ in two-sided t-test.

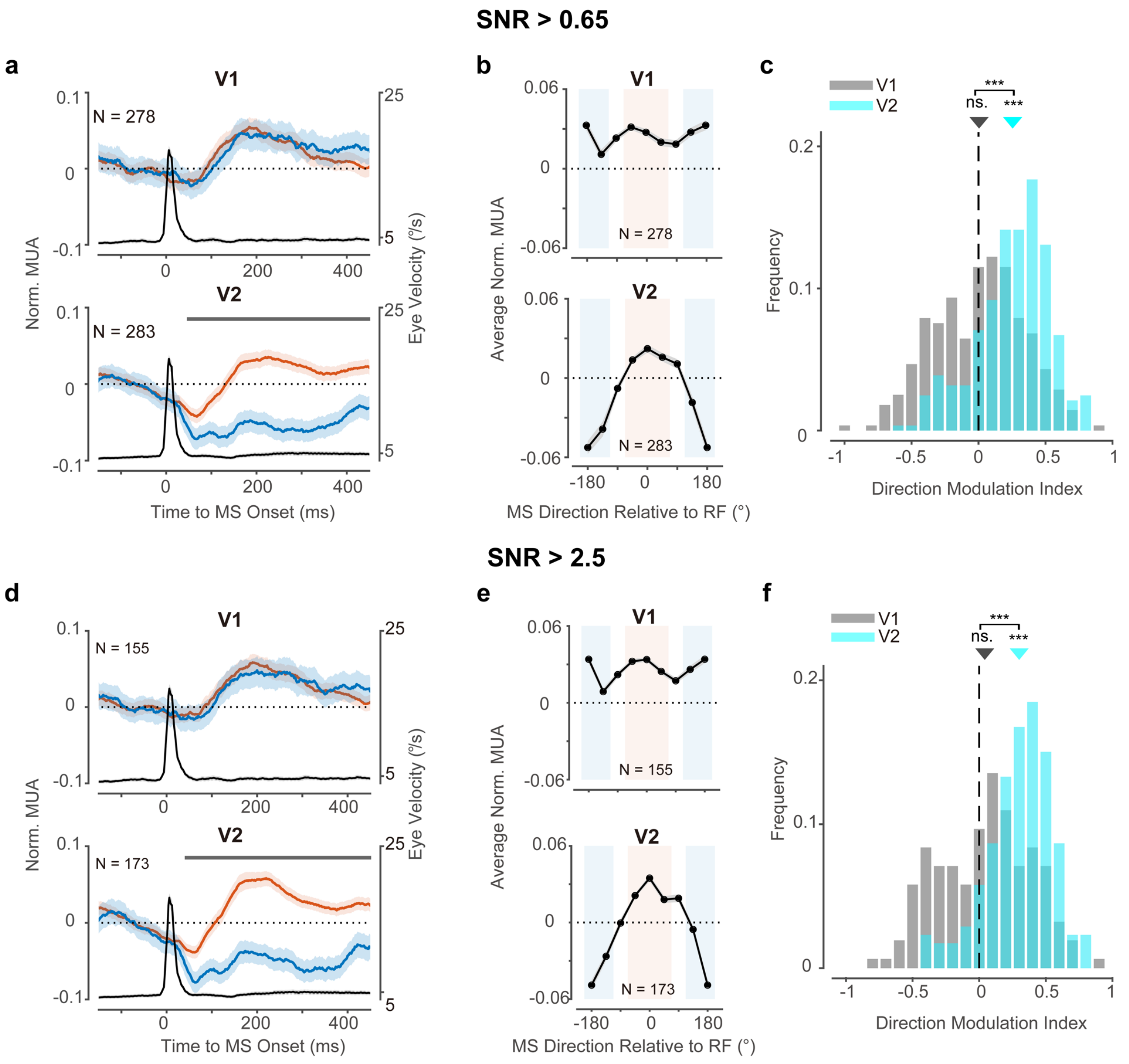

Supplementary Fig. 4 Microsaccade modulation on MUA in V1 and V2 with good channels

a Normalized firing rates (mean ± s.e.) around microsaccade in V1 and V2 after selecting channels with SNR > 0.65. **b** Microsaccade direction tuning for the averaged responses of V1 and V2 (mean ± s.e.) from the period 125~450 after microsaccades, red and blue shadow regions indicate two direction bins used for calculated direction modulation index in **c**. **c** Distribution of direction modulation strength in V1 and V2 (two-sided *t* test, '***': $p < 10^{-10}$, 'ns.': not significant). **d** Same as (**a**) but the units were selected by the criteria of 2.5. **e** Same as (**b**) for units with SNR over 2.5. **f** Same as (**c**) for units with SNR over 2.5. (two-sided *t* test, '***': $p < 10^{-10}$, 'ns.': not significant)

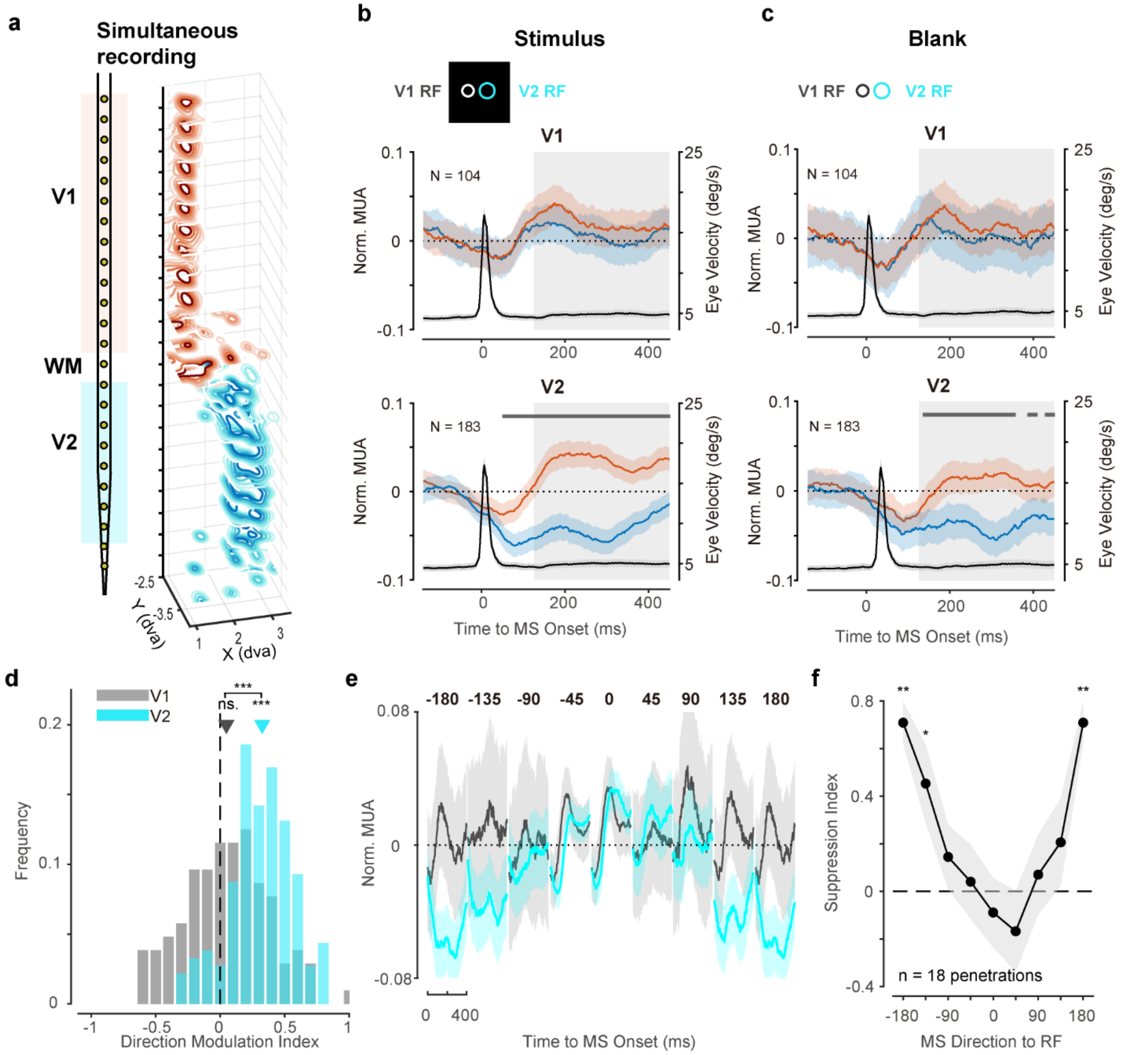

Supplementary Fig. 5 Direction-specific microsaccade suppression from simultaneous recordings in V1 and V2

a Simultaneous laminar recording with a multielectrode linear array (24 channels, V-probe) and stacked contour plots of receptive field mapped along each contact point in the probe from an example session. Region of V1 is marked by orange and V2 is marked by cyan. For all 18 sessions of simultaneous recordings, all layers of V2 and middle-to-deep layers of V1 were recorded with one probe. **b** Neuronal activity averaged across V1 units (top) or V2 units (down) (mean ± s.e.) in the two directions with a stimulus (4-6°) shown in the center of the receptive fields. Black curves are averaged eye velocity aligned by microsaccade onset. Gray bars indicate statistical significance between the two conditions (paired *t*-test with Bonferroni corrections). **c** Same as (**b**) but without any stimulus on

the screen with a grey background. **d** Distributions of direction modulation index (DMI) of V1 and V2 units (two-sided *t*-test, '***' indicates $p < 10^{-10}$, 'Ns.' indicates $p > 0.05$). The population distribution of DMI for V2 units was higher than DMI distribution for V1 units (two-sided *t*-test, V1 vs V2: $t(285) = 7.57$, $p = 5.21\times10^{-13}$; V1: $t(103) = 1.58$, $p = 0.12$; V2: $t(182) = 17.15$, $p = 7.51\times10^{-40}$). **e** Averaged MUA (mean ± s.e.) in V1 (gray) and in V2 (cyan) after microsaccades with 9 directions relative to RF locations of the recorded units. **f** Averaged suppression index (mean ± s.e.) across penetrations (n = 18) was tuned by microsaccade directions relative to RFs. Asterisks denote statistical significance (two-sided *t* test, '*': $p < 0.05$, '**': $p < 0.001$, after Bonferroni corrections for multiple comparisons).

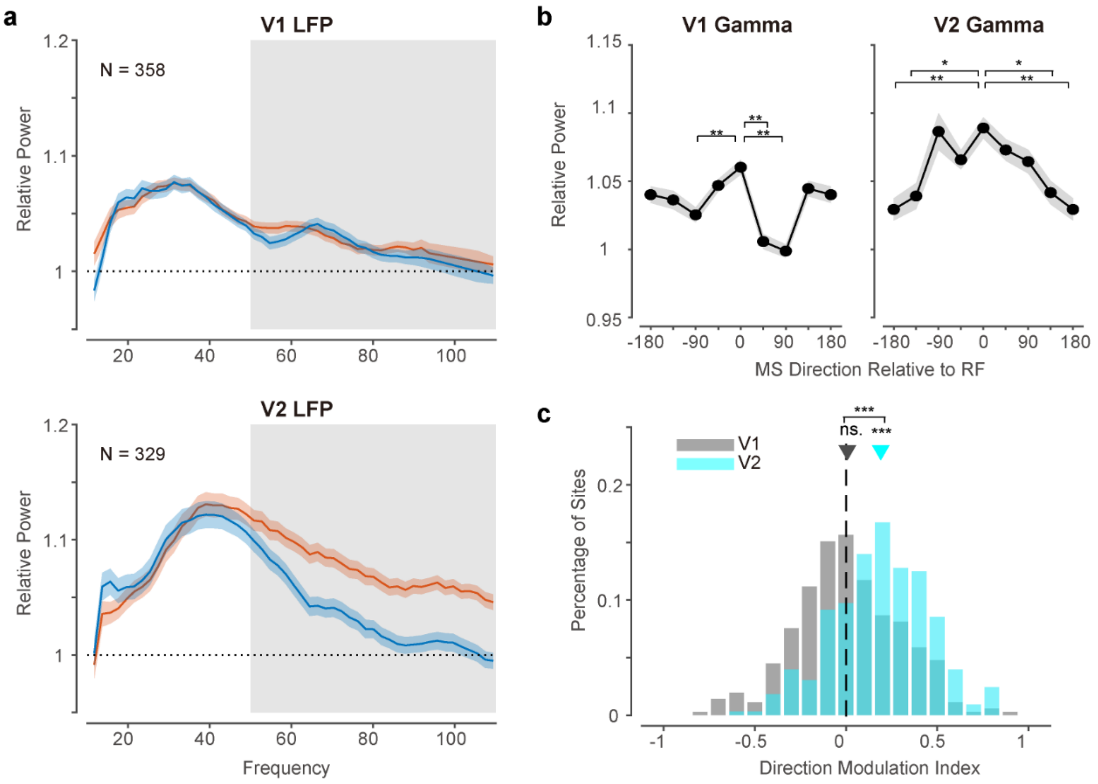

Supplementary Fig. 6 Direction-specific microsaccade modulation on local field potentials

a Power spectrum (mean ± s.e.) calculated from the LFP 250~600 ms after microsaccades in V1 and V2. The gray region indicates the gamma band (50~85 Hz). **b** Microsaccade direction tuning (mean ± s.e.) on averaged gamma power in V1 and in V2 (*Post hoc* comparisons of one-way repeated-measures ANOVA with Bonferroni corrections, '*': $p < 0.05$, '**': $p < 0.001$). Gamma power in V2 is tuned to relative direction between microsaccade and RF (*Post hoc* comparisons of one-way repeated-measures ANOVA with Bonferroni corrections, 0° vs. -180°: $p = 0.0001$; 0° vs. -135°: $p = 0.003$; 0° vs. 135°: $p = 0.0066$) **c** Distribution of direction modulation on gamma power in V1 and V2. Asterisks denote statistical significance (two-sided t test, '***': $p < 10^{-10}$, after Bonferroni corrections). Direction modulation in V2 is stronger than V1 (two-sided t test, V1 vs V2: $t(685) = 8.71$, $p = 2.26\times10^{-17}$; V1 vs 0: $t(357) = 0.49$, $p = 0.63$;V2 vs 0: $t(328) = 13.18$, $p = 4.06\times10^{-32}$).

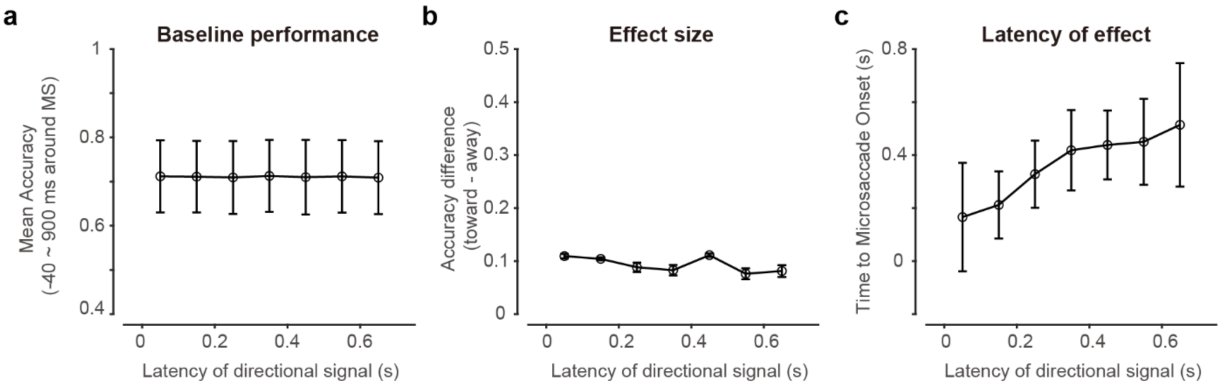

Supplementary Fig. 7 Simulated behavioral results changing with the latency of direction-dependent suppression.

a Independence between direction-specific discharge latency and averaged simulated accuracy across time after microsaccades from both the toward and away conditions (n = 20 simulations). Error bars denote SD across simulations. **b** Independence between discharge latency and effect size calculated as the difference in the accuracy from the toward and away conditions (n = 20 simulations). Error bars denote SD across simulations. **c** The relationship between latency of significant direction modulation on simulated behavior and discharge latency (n = 20 simulations). Error bars denote SD across simulations.

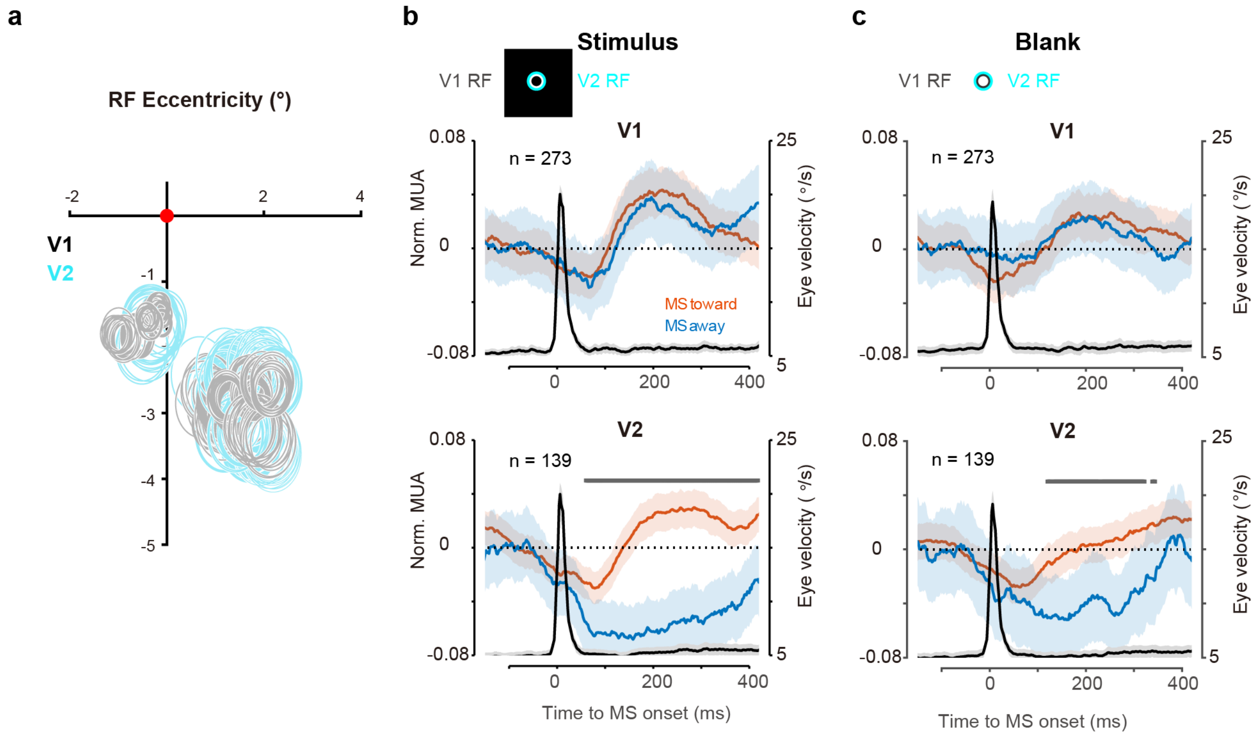

Supplementary Fig. 8 Microsaccade modulation in V1 and V2 with overlapping RFs

a RFs of V1 and V2 units were overlapping (in each animal) from 18 V1 sessions and 12 V2 sessions. **b** Normalized MUA (mean ± s.e., orange and blue lines in the left vertical axis) in V1 (top) and V2 (down) and averaged velocity of eye movement (the black line in the right vertical axis) around microsaccade onset. Gray bar on the top indicates the significant time period in the two-sided paired *t*-test with Bonferroni corrections. **c** same as **b** but under blank condition.

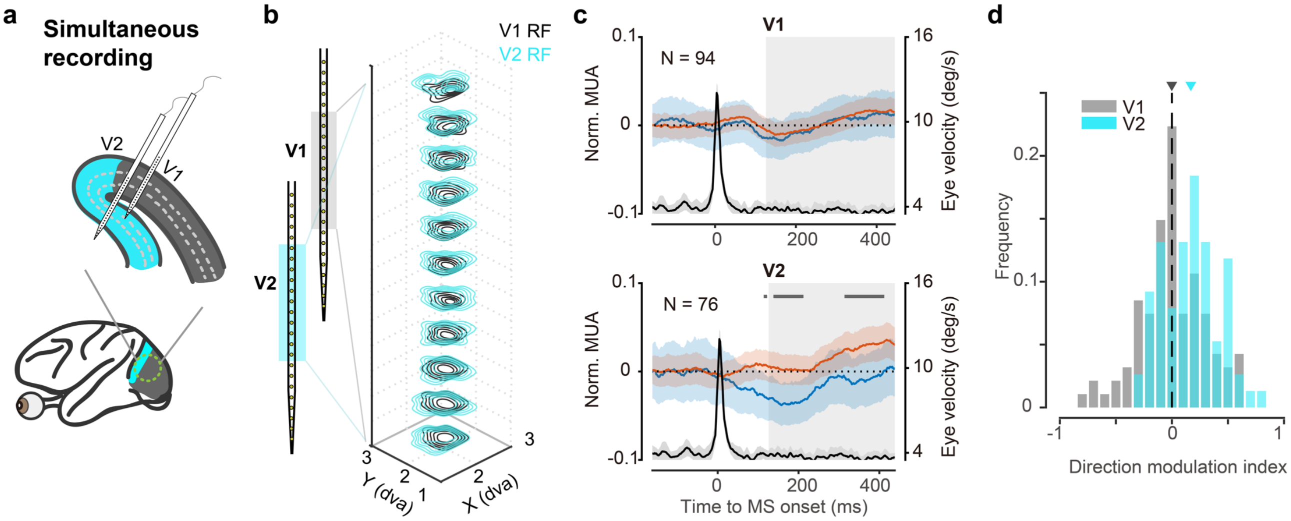

Supplementary Fig. 9 Simultaneous laminar recordings with two probes in V1 and V2 in monkey DS

a Simultaneous laminar recording in V1 and V2 with two linear probes in the control experiment. **b** The overlapping RFs of 11 channels from V1 and 11 channels from V2 in one example session. There were 7 sessions in total. **c** Normalized MUA (mean ± s.e.) in V1 and V2 (orange, MS toward; blue, MS away) and averaged velocity of eye movement (the black line in the right vertical axis) around microsaccade onset. Gray bar on the top indicates the significant time period in the paired *t* test with corrections for multiple comparisons. **d** Population distribution of direction modulation index of channels in V1 and V2 (two-sided *t*-test, V1 vs V2, $p < 10^{-10}$; V2, $p < 10^{-10}$; V1, $p > 0.05$).

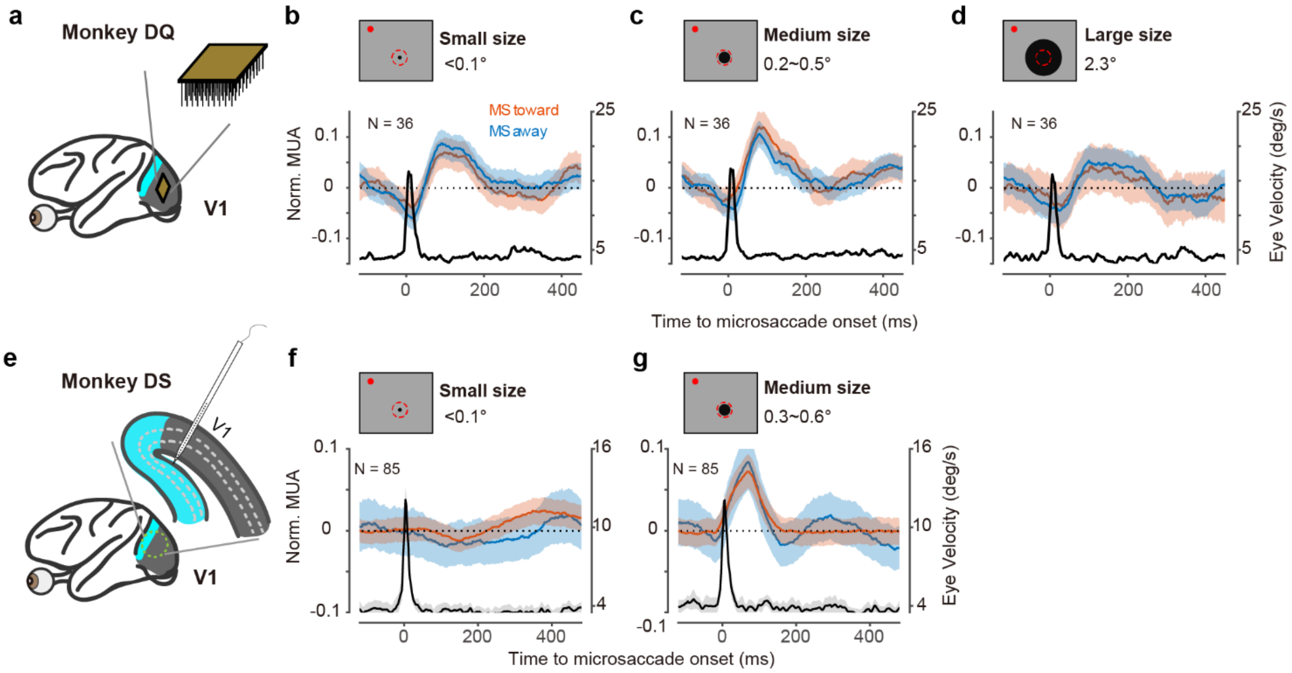

Supplementary Fig. 10 The control experiment in V1 with small stimuli.

a V1 recording with a Utah array in monkey DQ. **b** Normalized MUA (mean ± s.e.) in V1 with a small circle (<1°) represent on the center of RFs (orange and blue lines in the left vertical axis) and averaged velocity of eye movement (the black line in the right vertical axis) around microsaccade onset. **c** Same with b but the stimulus size was 0.2~0.5°. **d** Same with (**b**) and (**c**) but the stimulus size was 2.3°. **e** Laminar recording in V1 in monkey DS. **f** Same with (**b**) in monkey DS. **g** Same with (**f**) but the stimulus size was 0.3~0.6°.
