# Supplementary material for: V1-bypassing suppression leads to direction-specific microsaccade modulation in visual coding and perception
Source: Nat Commun. 2022 Oct 26;13:6366. doi: 10.1038/s41467-022-34057-3 (PMC9606005; doi:10.1038/s41467-022-34057-3)
Supplement: Supplementary file 3 — Reporting Summary [file 41467_2022_34057_MOESM3_ESM.pdf]

nature portfolio

Corresponding author(s): Dajun Xing

Last updated by author(s): Sep 6, 2022

Reporting Summary

Nature Portfolio wishes to improve the reproducibility of the work that we publish. This form provides structure for consistency and transparency in reporting. For further information on Nature Portfolio policies, see our Editorial Policies and the Editorial Policy Checklist.

Statistics

For all statistical analyses, confirm that the following items are present in the figure legend, table legend, main text, or Methods section.

n/a	Confirmed	
☐	☒	The exact sample size (*n*) for each experimental group/condition, given as a discrete number and unit of measurement
☐	☒	A statement on whether measurements were taken from distinct samples or whether the same sample was measured repeatedly
☐	☒	The statistical test(s) used AND whether they are one- or two-sided<br>*Only common tests should be described solely by name; describe more complex techniques in the Methods section.*
☐	☒	A description of all covariates tested
☐	☒	A description of any assumptions or corrections, such as tests of normality and adjustment for multiple comparisons
☐	☒	A full description of the statistical parameters including central tendency (e.g. means) or other basic estimates (e.g. regression coefficient) AND variation (e.g. standard deviation) or associated estimates of uncertainty (e.g. confidence intervals)
☐	☒	For null hypothesis testing, the test statistic (e.g. *F*, *t*, *r*) with confidence intervals, effect sizes, degrees of freedom and *P* value noted<br>*Give P values as exact values whenever suitable.*
☒	☐	For Bayesian analysis, information on the choice of priors and Markov chain Monte Carlo settings
☒	☐	For hierarchical and complex designs, identification of the appropriate level for tests and full reporting of outcomes
☐	☒	Estimates of effect sizes (e.g. Cohen's *d*, Pearson's *r*), indicating how they were calculated

Our web collection on statistics for biologists contains articles on many of the points above.

Software and code

Policy information about availability of computer code

Data collection	Raw data were acquired by a 128-channel system (Blackrock Microsystems).
Data analysis	Custom code was written using MATLAB (v.2018b) which is available as https://github.com/yujie1447/NC_Microsaccade.

For manuscripts utilizing custom algorithms or software that are central to the research but not yet described in published literature, software must be made available to editors and reviewers. We strongly encourage code deposition in a community repository (e.g. GitHub). See the Nature Portfolio guidelines for submitting code & software for further information.

Data

Policy information about availability of data

All manuscripts must include a data availability statement. This statement should provide the following information, where applicable:

- Accession codes, unique identifiers, or web links for publicly available datasets
- A description of any restrictions on data availability
- For clinical datasets or third party data, please ensure that the statement adheres to our policy

The data supporting the findings of this study are available as https://github.com/yujie1447/NC_Microsaccade. Source data are provided with this paper.

Human research participants

Policy information about studies involving human research participants and Sex and Gender in Research.

Reporting on sex and gender	N/A
Population characteristics	N/A
Recruitment	N/A
Ethics oversight	N/A

Note that full information on the approval of the study protocol must also be provided in the manuscript.

Field-specific reporting

Please select the one below that is the best fit for your research. If you are not sure, read the appropriate sections before making your selection.

☒ Life sciences ☐ Behavioural & social sciences ☐ Ecological, evolutionary & environmental sciences

For a reference copy of the document with all sections, see nature.com/documents/nr-reporting-summary-flat.pdf

Life sciences study design

All studies must disclose on these points even when the disclosure is negative.

Sample size	No statistical methods were used to predetermine sample sizes but our sample sizes are chosen based on our previous publications (Yang et al., 2022). We did electrophysiological recording on three monkeys for the formal experiment, and one of them for the control experiment. There are 358 recording sites in V1 (23 sessions) and 329 recording sites in V2 (32 sessions) for the formal experiment, 104 simultaneous-recording sites in V1 from V2 sessions (18 sessions) and 73 recording sites in V2 for the control experiment of surface motion. In the behavioral dataset, we collected responses from two monkeys across 19 and 21 formal sessions and 6 threshold-testing sessions.
Data exclusions	No data were excluded in the initial analyses.
Replication	Monkeys were trained and tested over a period of more than a month and produced consistent behavioral results in early and late phases. We replicated our findings of direction-specific microsaccade modulation in V2 in all three monkeys so we think the findings are reproducible.
Randomization	No experimental groupings were used in this study.
Blinding	Blinding is not relevant because no grouping was performed.

Reporting for specific materials, systems and methods

We require information from authors about some types of materials, experimental systems and methods used in many studies. Here, indicate whether each material, system or method listed is relevant to your study. If you are not sure if a list item applies to your research, read the appropriate section before selecting a response.

Materials & experimental systems

n/a	Involved in the study	
☒	☐	Antibodies
☒	☐	Eukaryotic cell lines
☒	☐	Palaeontology and archaeology
☐	☒	Animals and other organisms
☒	☐	Clinical data
☒	☐	Dual use research of concern

Methods

n/a	Involved in the study	
☒	☐	ChIP-seq
☒	☐	Flow cytometry
☒	☐	MRI-based neuroimaging

Animals and other research organisms

Policy information about studies involving animals; ARRIVE guidelines recommended for reporting animal research, and Sex and Gender in Research

Laboratory animals	Four male adult rhesus monkeys (Macaca mulatta, 5-7 years, 6-8 Kg).
Wild animals	The study did not involve wild animals.
Reporting on sex	All Four monkeys are male.
Field-collected samples	The study did not involve samples collected from field.
Ethics oversight	All procedures were conducted in compliance with the National Institutes of Health Guide for the Care and Use of Laboratory Animals, and were approved by the Institutional Animal Care and Use Committee of Beijing Normal University.

Note that full information on the approval of the study protocol must also be provided in the manuscript.
